# Obstacles and Enablers on the Way towards Integrated Physical Activity Policies for Childhood Obesity Prevention: An Exploration of Local Policy Officials' Views

**DOI:** 10.1155/2016/5739025

**Published:** 2016-09-07

**Authors:** Anna-Marie Hendriks, Jolanda M. Habraken, Stef P. J. Kremers, Maria W. J. Jansen, Hans van Oers, Albertine J. Schuit

**Affiliations:** ^1^Academic Collaborative Centre for Public Health Limburg, Regional Public Health Service, Geleen, Netherlands; ^2^Department of Health Promotion, School of Public Health and Primary Care (CAPHRI), Maastricht University, Maastricht, Netherlands; ^3^Scientific Center for Care and Welfare (TRANZO), Tilburg University, Tilburg, Netherlands; ^4^Department of Health Promotion, School of Nutrition and Translational Research in Metabolism (NUTRIM), Maastricht University, Maastricht, Netherlands; ^5^Department of Health Services Research, School of Public Health and Primary Care (CAPHRI), Maastricht University, Maastricht, Netherlands; ^6^National Institute for Public Health and the Environment, Bilthoven, Netherlands; ^7^Department of Health Sciences and EMGO Institute for Health and Care Research, Vrije Universiteit, Amsterdam, Netherlands

## Abstract

*Background*. Limited physical activity (PA) is a risk factor for childhood obesity. In Netherlands, as in many other countries worldwide, local policy officials bear responsibility for integrated PA policies, involving both health and nonhealth domains. In practice, its development seems hampered. We explore which obstacles local policy officials perceive in their effort.* Methods*. Fifteen semistructured interviews were held with policy officials from health and nonhealth policy domains, working at strategic, tactic, and operational level, in three relatively large municipalities. Questions focused on exploring perceived barriers for integrated PA policies. The interviews were deductively coded by applying the Behavior Change Ball framework.* Findings*. Childhood obesity prevention appeared on the governmental agenda and all officials understood the multicausal nature. However, operational officials had not yet developed a tradition to develop integrated PA policies due to insufficient boundary-spanning skills and structural and cultural differences between the domains. Tactical level officials did not sufficiently support intersectoral collaboration and strategic level officials mainly focused on public-private partnerships.* Conclusion*. Developing integrated PA policies is a bottom-up innovation process that needs to be supported by governmental leaders through better guiding* organizational processes* leading to such policies. Operational level officials can assist in this by making progress in intersectoral collaboration visible.

## 1. Introduction

Childhood obesity rates have risen dramatically in the last decades. In 2013, 23.8% of boys and 22.6% of girls in developed countries were overweight or obese, while in developing countries, 12.9% of boys and 13.4% of girls were overweight or obese [1–4]. Even though obesity has plateaued in some countries, projections to 2030 indicate rates will continue to rise in most countries [5–7].

Low physical activity (PA) levels are an important driver of the childhood obesity trend [8–12]. PA also contributes to children's academic achievement and cognition [13, 14]. Unfortunately, few children meet the recommended guidelines of 60 minutes of PA per day [15–18]. For example, in the United States, only 42% of children between 6 and 11 years of age meet the recommended guidelines [19], and in Netherlands, only 19% of children are in primary school [20].

Due to these low PA levels among children, initiatives to stimulate PA are implemented globally [17, 18]. So far, only few have been successful since the determinants of PA have a “wicked” character and policies to address this are often not implemented [21, 22]. PA is affected by many interrelated determinants and is influenced by many different factors, such as social and physical environment as well as psychosocial factors and habits and hence there is no single solution to stimulate PA that applies in all circumstances [22, 23]. Wicked is referring to the multicausal nature and social complexity (i.e., involving a wide range of actors) of the problem. In contrast to “tame” problems, which might be technically complicated, wicked problems are also socially complex. Therefore wicked problems cannot be tightly defined and solved by linear analytical approaches but require more innovative and collaborative (intersectoral) problem solving approaches [22–24].

This wicked character implies that, in order to reverse this trend, practitioners and policy makers need input from sectors and stakeholders both inside and outside the public health domain, such as spatial planning, education, safety, social affairs, employment, and sports organizations, using an “integrated” PA approach [22, 25]. In Netherlands, an example of a program that supports the development of such an approach is “Youth on a Healthy Weight” (in Dutch known by the acronym “JOGG”) [26].

It is assumed that “integrated PA policies” enable and improve long-term commitments [12, 27, 28]. Such integrated policies are characterized by two criteria: (1) the policies include an appropriate mix of interventions that ensure that motivation, capability, and opportunity of the target population are improved in such a way that it stimulates physical activity and (2) the policies are implemented by the relevant policy sectors from different policy domains (i.e., intersectoral collaboration) [29]. Examples of integrated PA policies include increasing active travel options (e.g., through cycling paths), making a range of physical activities available for children of all socioeconomic groups, health-enhancing urban planning policy, and promoting the use of active travel options by parents (e.g., [9, 12, 28]).

In many countries, local governments are in a unique position to develop integrated PA policies [25]. Within Dutch local governments, three levels of actors and several sectors are involved in developing integrated PA policies. At the strategic level, the relevant alderman (who are in Netherlands not members of the city council and have separate responsibilities), the mayor, and the municipal council are involved, at the tactical level, heads of municipal departments, and at the operational level the administrative system, consisting of civil servants. With regard to the different policy sectors, officials from the transport, urban planning, and financial domains have the power and financial means to develop and implement policy on spatial planning, for example, to create a health stimulating environment [30].

These policies can affect, for example, street lighting, speed limits in residential areas, sidewalks, and school environments [31]. Public health issues fall under the responsibility of officials within the social domains such as welfare, health, and education, primarily supported by Public Health Services (PHSs) [32]. Since PA is affected by issues in both domains, “intersectoral collaboration” (ISC) is required. For example, to develop safe and attractive walking and cycling networks in local neighborhoods to stimulate active transport, officials of health, spatial planning, transport, and nature (officials charged with educating citizens about their natural environment and supporting the maintenance of the natural environment) need to collaboratively explore how active transport options between the home and school environment can be increased.

Unfortunately, the development of integrated PA policies within governments has proven to be difficult [23, 25, 33]. Previous studies showed a wide range of factors that block officials' way or hinder progress towards ISC. Examples of such obstacles for ISC are limited sharing of policy goals, a lack of coaching of officials by managers, and low motivation to learn new ISC skills [33–35]. Furthermore, limited financial resources, limited evidence for policy effectiveness [35], low feasibility of environmental policy measures [36, 37], and framing of obesity as an individual health problem may hamper the development of integrated policies (e.g., [38]). Some authors have suggested that obesity might become amenable to broad policy solutions if those problems are framed in systemic terms (e.g., [39]). This means obesity is regarded as not only an individual but also an environment responsibility.

All these obstacles are interrelated. Therefore, experts not only describe PA as a wicked problem but also describe the process towards integrated PA policies as a* “messy affair that does not neatly stick to stages”* (e.g., [40, 41]). For example, policy officials within the local government often know that even if a policy alternative for PA was analyzed thoroughly and is well-planned and put on the agenda (i.e., debated), it might not be implemented (e.g., due to shift in power) [27].

The wicked nature of integrated physical activity policies for childhood obesity prevention implies that it is important to understand the perspective of those involved in it. In Netherlands, these are formally the local policy officials, supporting institutions (e.g., JOGG), other levels of government, representatives of settings (e.g., school directors, child-care managers), and recently also private partners and citizens [34, 42, 43]. Previous studies extensively explored the views of officials regarding ISC and integrated public health policy at the national government, from the point of view of private partners or citizens, within one sector or setting, focusing on one policy measure only (e.g., soft-drink taxation) (e.g., [25, 33, 34, 36, 44–56]). However, fewer studies (e.g., [44]) captured in-depth view of local government officials from different policy sectors (i.e., expertise fields) (e.g., [45, 57]) and applied an explicit theoretical perspective and thereby contribute to theory development. Since capturing these perspectives might provide a broader view on the landscape in which such policies are developed, we expect this can yield new insights that can take the development of integrated PA policies a step further. Therefore, we aimed to answer the following research question:* “Which obstacles and enablers do local policy officials perceive during the development of integrated physical activity policies?”*

## 2. Methods

### 2.1. Theoretical Framework

**Figure 1:**
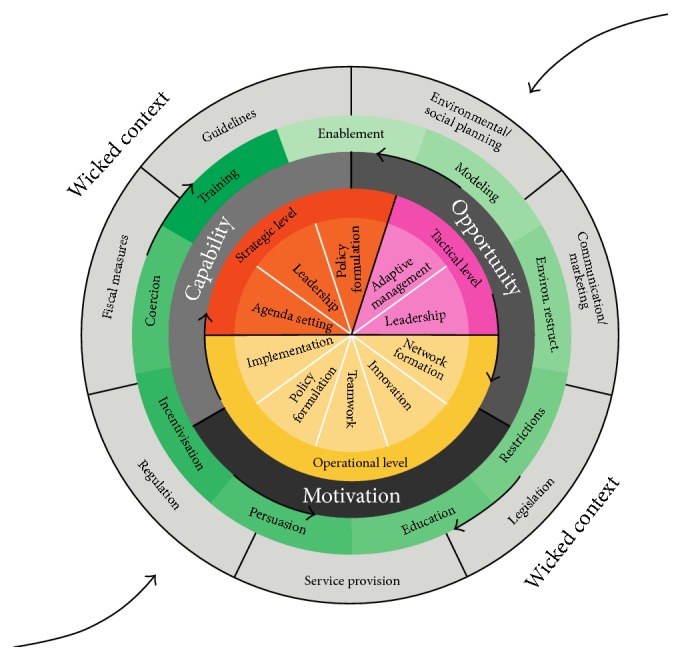
The Behavior Change Ball. The Behavior Change Ball consists of circles that reflect organizational behaviors, actors within three hierarchical levels, determinants of organizational behaviors, interventions, and policies or programs. Policies or programs enable interventions, and determinants are necessary for each of the organizational behaviors that are related to actors at the three hierarchical levels, that is, operational, tactical, or strategic level.

**Figure 2:**
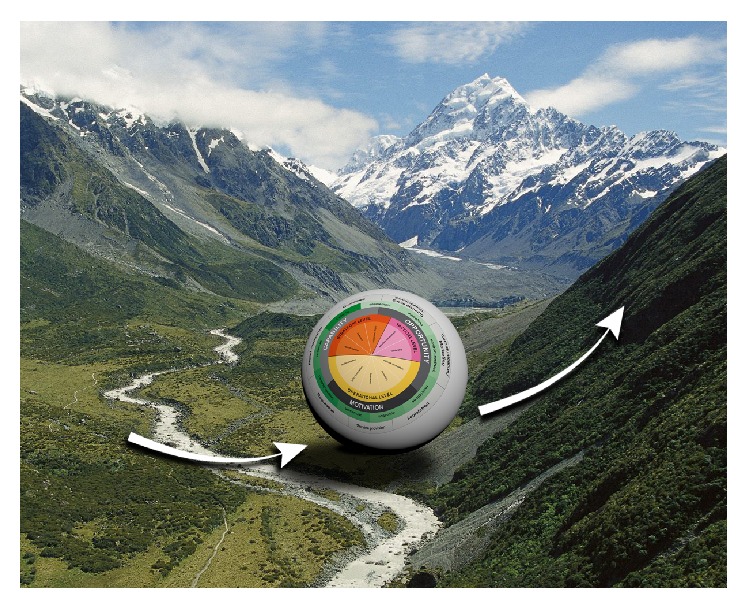
The Behavior Change Ball moving through a landscape. The proposed relationships between the theoretical concepts from the Behavior Change Ball are best illustrated by the metaphor of a ball moving through a landscape [49].

To capture obstacles within the policy process, theories need to be applied that capture the wicked, multilevel, and incremental nature of elements in the process [27]. One framework that explicitly recognizes this character is the Behavior Change Ball (BCB) (Figures 1 and 2). The BCB was specifically developed to explain the development of “integrated” public health policies. Although there exist a plethora of frameworks for policy development, the BCB is (so far) the only framework specifically developed to explain the development of “integrated” public health policies [49].

The framework is developed to capture and understand organizational behaviors (OBs) of local policy makers at strategic, tactical, and operational levels that are essential for integrated policy. Ten OBs are specified and related to capability, opportunity, and motivation (COM) and nine intervention functions and seven policy categories are formulated (Figure 1). Although the BCB framework in itself does not explain or predict OBs, it aims to capture obstacles that can explain variation in policy development. Therefore, the BCB was considered the most appropriate framework for the current study.

### 2.2. Study Setting

Our study setting consisted of three Dutch municipalities (i.e., cases) that had just finished their policy plans (primarily policy goals) and, as seen in their policy documents, intended to develop integrated public health policies. They were similar in size and were considered “large” municipalities for Dutch standards; they had around 180,000 inhabitants, of which approximately 10% of the youth between 0 and 18 were overweight and within low SES neighborhoods approximately 25% were overweight.

### 2.3. Data Collection

Semistructured interviews were chosen to explore complex obstacles that were affecting the development (operationalizing policy goals into concrete policy measures) of integrated PA policies. Interviews have ability to collect in-depth experiences, explore a topic that has not been extensively studied before, and can elicit more nuanced aspects of the development of integrated public health policies than by quantitative methods. We chose to conduct semistructured interviews because we were interested in a particular topic but also provide the opportunity for new topics to emerge during the interview.

The interview topic list was collaboratively decided by the two researchers and refined with sensitizing concepts after each interview. Questions focused on experiences during the development of integrated PA policies and on the development of integrated public health policies in general. We chose to focus not only on the obstacles encountered during the development of PA policies but also broader on integrated public health policies in general. This was expected to yield a broader view on the context in which such policies are developed. Prior to each interview, interviewees received information about the study and signed an informed consent form. Two researchers (Anna-Marie Hendriks and Jolanda M. Habraken) conducted the interviews. This was decided since it could enable reflection on interviews. Both researchers positioned themselves as interviewers employed by a public health department within the university. The researchers had not seen most nonhealth officials prior to the interview, but with health officials some contacts were present approximately three months prior to the interviews (mainly through attending meetings).

### 2.4. Sampling

We purposively sampled a heterogeneous (situated within different policy sectors and within different municipalities) interview sample. First, we identified key informants in each case and asked them to refer to other officials with whom they collaborated in general and for the prevention of childhood obesity specifically. From this initial sample, we purposively selected one representative of each policy sector. In this way, we aimed to obtain a representative sample of those that were involved in developing integrated public health policies. The ultimate sample (Table 1) included 15 officials who were (primarily) involved or responsible for 10 different sectors in three cases. In case 1 we interviewed seven respondents, and in cases 2 and 3 we interviewed 4 respondents. Most respondents were situated at the operational level as policy advisors and one was situated as policy implementer. Two respondents were leading a project as well and therefore considered partially situated at tactical level. Because they were not officially situated in a management function (e.g., as a head of the department), we primarily considered them to be operational level policy officials. Two respondents were situated at the strategic level: one council member and one aldermen.

### 2.5. Data Analysis

We applied a framework approach to deductively analyze our data. This approach was considered suitable since the objectives and aim of the study were set in advance [58]. We started deductively coding obstacles within the OB (policy formulation (operational and strategic), network formation, innovation, teamwork, adaptive management, leadership (strategic and tactic), agenda setting, and implementation) in the transcripts from case 1. Within each OB, we coded COM factors. The OBs that included most obstacles were regarded as “obstacles” and compared with our findings in cases 2 and 3. Our data analysis was ended after “theme saturation,” when no new obstacles were found [59]. We considered this method appropriate for the current study since our aim was to explore a wide range of obstacles among a heterogeneous interview sample within a relatively short period. The interviews were coded by two interviewees. If we detected inconsistencies between the codes, these were discussed by the two coders and together the most appropriate code was decided on. Analysis was thus split but discussed afterwards to reach consensus. In all cases the coders were able to reach consensus.

## 3. Findings

Interviewees mentioned obstacles in a wide range of OBs. We will now focus on the five OBs in which most obstacles were found: operational level policy formulation, teamwork, adaptive management, strategic leadership, and agenda setting.

### 3.1. Obstacle 1: Operational Level Policy Formulation

Interviewees said the intention to develop integrated PA policies was formulated on paper, but in practice it was more common to develop policies within one sector. Sports officials, for example, developed their own sport policy, the public health officials developed their own health policy, and the nature officials developed their own nature policy. Only in case 2 was it more common to develop one policy document for all sectors together. Interviewees in this case explained this facilitated ISC since they had shared goals. Moreover, one interviewee in this case added that previously the policy documents of each sector would not receive much attention after it was accepted by the council. So, she regarded the development of one document as a positive development.

Furthermore, all interviewees emphasized that developing more “integrated” PA policies was very much dependent on individuals. In Netherlands there is an increasing trend in local government to work in teams with high levels of autonomy (“self-governing” teams). However, not all officials were positive about this trend. Even though all interviewees valued autonomy, they noted that the lack of time made it sometimes impossible to invest in the development of integrated PA policies. One official from case 1, for example, said that she had to write the public health policy in two weeks. This limited her to seek contacts with officials from other sectors. This interviewee said she would have welcomed more time to write her policy plan and also another interviewee from this case noted this obstacle:

*At this moment [during the development of the policy plan] I really don't have sufficient time. When brainstorming with colleagues [about her policy plan], all sorts of ideas pop up. However, often I try not to do much with these ideas since I would need to explore a whole new territory*. (case 1, official from Nature)

Interviewees in this case added that it was sometimes difficult to develop integrated PA policies, since it was not always clear where policy objectives converged:

*I think that the objectives of sport, youth, education, youth health care are very often similar. And they should*…*However, when implementing these policies it is easy to see this [similarity in objectives], however, sometimes you do not notice it at a higher level [by operational level officials responsible for policy formulation].* (case 1, official from Sport)

### 3.2. Obstacle 2: Teamwork

All interviewees recognized that the development of integrated PA policies required teamwork between officials from health and nonhealth sectors (ISC). Interviewees said it was common to start with exploring which partners* within *their own sector could be involved. For example, public health officials would first seek collaboration with officials in their own “sector” such as youth, education, and nature (but outside their own “program”). After that they would seek collaboration with leaders from other sectors and programs within those other sectors, such as spatial planning and environment. Interviewees therefore explained organizational structures with sectors including more programs could stimulate ISC (one sector could include several programs). Interviewees added to this that limited sharing of budgets could be an obstacle for teamwork and thus more sharing of budgets would help. Indeed, interviewees in case 2 expressed that the budget for “citizens participation” facilitated that ISC was established.

Furthermore, interviewees frequently referred to differences in department cultures as obstacles for ISC. According to them, these were grounded in different “belief systems” in the physically oriented sectors (e.g., street lighting, speed limits in residential areas, and sidewalks) and socially oriented sectors (e.g., health education, safety, and sustainability). Interviewees explained that while socially oriented sectors focused on the why of certain actions (e.g., explaining why a new playground is needed) and were often generalists, physically oriented sectors would skip this “why question” and immediately work on the “what” (e.g., starting to design a new playground) and were more often specialists. Interviewees said this sometimes created a language barrier that would hamper effective communication. In case 3, one official explained that these differences sustained due to the recruitment strategy of managers in each sector.

Many interviewees mentioned that if such structural and cultural obstacles could be overcome, individual characteristics would be decisive for actual teamwork. They said it was often easier when officials were enthusiastic, personally knew officials from previous projects, had positive experiences during previous collaboration, had an “open mind,” had the ability to think holistically (recognizing interdependencies between sectors), were generalists, viewed other sectors as “vehicles” to achieve ones' own goals (e.g., stimulating cycling to reduce greenhouse gas emissions), had work experience in health and nonhealth sectors, and had boundary-spanning skills:

*this is what you would have liked [ISC between public health and spatial planning officials] *…*but, you still often notice it depends on individuals, people want to collaborate, but often I still function as the ‘key'. This is because I very well know what the others [within spatial planning] are busy with*…*they [refers to the public health and spatial planning officials] know somehow what the others are busy with, but not enough to go there with a really specific question. So, often I say: ow yes, since I sit within the same office [of the spatial planning official with whom public health officials would like to collaborate], I will ask her. Thus, that is how it in practice often works.* (case 1, official from Education who previously worked in spatial planning)

Interviewees often added that specialists were sometimes not able to see how specialism could be overcome and not able to communicate and collaborate* efficiently* and thus could be an obstacle for ISC. They also added that efforts to implement team-building interventions were taken but that the high workload, many part-time workers, and insufficient follow-up of such activities did not have the intended effect.

### 3.3. Obstacle 3: Adaptive Management

It was interesting to find that interviewees almost never spontaneously mentioned the role of their manager. Only when the interviewer explicitly asked them about this did they express their role. This might be explained by the comment of one interviewee:

*I think the head of the department is not that much involved in the process [of stimulating team work to develop integrated policy]. But the team leader is*…*she is really open for it*…*we are working according to the principles of self-governance*…*so the effect of the leaders on this process [of developing integrated policy] is actually very small*. (case 1, official responsible for policy implementation)

Interviewees added that they wanted to be incentivized for ISC and that their manager would help them in seeing how several programs within government could be connected. Some interviewees carefully expressed that they doubted if managers themselves had the motivation to incentivize ISC or had the capability to make such connections. It sometimes seemed that the manager focused on serving political goals. These goals could not be achieved by investing in ISC since this was considered a long-term internal organizational process. Interviewees added that managers sometimes had different opinions on how to stimulate ISC and thus little action was taken. Some interviewees mentioned that current managers were not from the health sector themselves; their expertise was mostly based on previous work experience in* one* of the 16 sectors and making connections was also difficult for them. Most interviewees expressed this was unfortunate since new organizational structure created clear opportunities to make such connections (work was increasingly based on themes rather than sectors).

### 3.4. Obstacle 4: Strategic Leadership

Interviewees explained that aldermen were interested in collaboration with private partners or citizens and described strategic leaders as “networkers”; they were modeling collaboration but were not seen as a role model for ISC within their own organization. Interviewees said that strategic level actors acknowledged the multicausal nature of obesity, but despite this, childhood obesity was still seen as an issue that could be solved with private stakeholders and not with nonhealth policy sectors* within* government. Interviewees explained this limited shared ownership of the problem by the national government focuses on partnerships with private partners and citizens participation.

Interviewees also added that it would depend on each new coalition if leadership will impose an obstacle for the development of integrated PA policies. For example, one interviewee from case 2 explained that at least three to four aldermen need to accept a proposal before actual PA policy can be made. Interviewees said that removing this obstacle was considered impossible due to the political nature of their work. However, within case 2, policy officials from the operational level were able to convince the strategic level leaders to invest in ISC and integrated policy and therefore received resources for this.

### 3.5. Obstacle 5: Agenda Setting

All interviewees mentioned the childhood obesity problem was recognized as a policy issue and most governmental actors understood that the environment was an important driver of this problem:

*attention for overweight is not that explicitly as in the past, but much more in an indirect way, we work a lot more on conditions that lead to overweight*…*also developing physical-activity-friendly neighborhoods. Trying to persuade people to get active and in that way prevent overweight*. (case 2, official from Public Health)

However, interviewees said that, despite this notion, the development of PA policies would not naturally come to the fore as a “solution” for this problem. They said that the urgency to prevent childhood obesity by developing PA policies needed to be increased. This could be achieved by consorted effort of operational level policymakers from both health and nonhealth sectors. Interviewees also noted that prevention in general was not a topic by which a politician could “score,” since the pay-offs for prevention would only potentially become visible in the long term. The current political liberal trend and budget cuts made investing in prevention politically sensitive; politicians were seen as reluctant to intervene in food or physical activity choices. Investing in “resource-demanding” and “autonomy-reducing” content of integrated PA policies was seen as unattractive.

## 4. Discussion

Local governments are increasingly stimulated to develop integrated PA policies. In developing such policies they need to overcome many “wicked” process obstacles. To develop support, we aimed to obtain in-depth insight in these obstacles from the perspective of local policy officials from health and nonhealth sectors. Our main findings indicate obstacles are mainly encountered during (operational level) policy formulation, teamwork, adaptive management, strategic leadership, and agenda setting. We will now discuss these obstacles and how they might be overcome.

First of all, it still does not seem “business as usual” to develop one policy document that integrates different policies. Often, sports sector officials develop “sport policy,” public health officials develop “physical activity policies,” and spatial planning officials develop “zoning policies” (policies that regulate the size, type, structure, and use of land or buildings in designated areas). So, even though “integration” is included in most governmental missions, and organizational structures were designed to enable ISC, the actual practice of integrated policy remains challenging. This was also described by previous studies (e.g., [33–35]). Budgets, subsidies, responsibilities, national standards, and legislation are often distributed along sectoral lines. Obviously, this obstacle seems very hard to overcome if governments are not investing in defining broader goals across sectors with one joint budget. In line with Hendriks et al. [35] and Storm et al. [55], we therefore recommend defining government wide public (health) goals at both the local and national level and increase early engagement with stakeholders outside the health sector (e.g., [53]). For example, “sustainability” might be a goal that allows involvement of officials representing finances, sports policy, public health policy, spatial planning policy, mobility policy, nature, and environment. From the health perspective, the interest would be to stimulate PA, while from the spatial planning perspective the interest would be in improving zoning policies, and from the mobility perspective the interest could be to develop well-connected cycling networks. Contribution mapping might be a tool that supports such alignment of goals [60].

Secondly, it does not seem easy to work in teams composed of officials with health and nonhealth backgrounds due to the bottom-up and innovative character of such teamwork and the scarce top-down guidance. Borins [61] also found that innovations in government usually start at the operational level but need to be supported by management and endorsed at the top. It seemed that “boundary-spanning skills” [62] were key in establishing teamwork; officials need to be motivated and able to look outside their comfort zone [63]. For example, if public health officials want to develop more playgrounds, they need to proactively approach colleagues from spatial planning and discuss possibilities. Therefore, ISC requires overcoming structural and cultural obstacles [64], such as the lack of time and different cultures in each sector. This was also found in previous studies (e.g., [35–37, 41, 50]). We therefore recommend exploring how to improve “boundary-spanning skills” and set structural conditions so it is easier to invest in ISC within teams. For example, changing the recruitment strategy, rotate soft and hard domain officials in each other's work environment, giving rewards for ISC, and remove asymmetric incentives, providing “venture” capital (e.g., budget specific to develop innovations) [62, 65]. Besides, instead of putting health at the core of integrated policy, it is worth to reframe health as a vehicle to achieve other sector's policy goals, for example, by framing active transport (e.g., by cycling or by walking) as a means to achieve sustainable environments.

Thirdly, it seems that “passive” managerial commitment for the development of integrated PA policies is insufficient. Although several sectors shared a manager who would be responsible for more than one sector and thus knows elements shared by the sectors, these managers seemed reticent to proactively coach officials in this. Managers did not provide incentivizes for ISC or help officials see how several programs within government are connected. Even though autonomy seems important for officials [35], without any structure and leadership, autonomy can bring a team down if officials do not have the same goals [66]. Since in government each policy sector has its own goals, the team would first need to invest time in finding a shared goal. If there is no time for finding such a shared goal and managers do not assist in this, autonomy seems an ineffective way to optimize teamwork. Management of wicked public health problems requires an “adaptive” approach, with an emphasis on “learning by doing” [40, 41], for example, learning from evidence for integrated PA policies and utilizing this evidence in experiments [65]. To facilitate this, process outcomes need to become visible and experiences during implementation may be used as a way to show their leaders the importance of investing in processes. Researchers can assist in this by responsive or participatory process evaluations during the implementation process and apply comprehensive approaches to the evaluation of integrated policies [67]. Additionally, implementers might provide input for officials that develop more “integrated” policies; for example, they can easily show that a newly created playground is not used by children since the sports policy implements a program to get children to “their” sport facilities. Therefore, they provide a motivator for more ISC at the policy level.

Fourthly, it seems that strategic leaders are focused more on private partners (e.g., food industry) and citizens and on “quick wins” than on collaboration with other sectors within their own government. It seemed, for example, more attractive for them to open a new playground than to invest in policy changes that make sure that each neighborhood has a safe playground. This focus may be related to the emphasis of the national government on public-private partnership and limited financial resources. To overcome this obstacle, leaders need to be made cognizant that their role is to promote internal ISC as well and understand partnerships with private partners are not the only way forward [43, 68].

Fifthly, it seems that childhood obesity prevention appears on the agenda, but the organizational changes that contribute to the development of integrated PA policies are not. This seems related to the recent economic crisis, neoliberal trend, the time-horizons of most politicians, and the framing of the problem. In the aftermath of an economic crisis, economic growth might be an overriding argument and affect the actions and decisions strategic actors take. Intervening in markets and private lives of citizens, confronting powerful marketing activities and lobbies by private companies are politically unattractive (e.g., [38]). Therefore, self-regulation, public-private partnerships, and citizen participation might attract more attention [43, 68], even though these are also challenging (e.g., [42]). Moreover, investments in ISC and prevention do not often result in visible results (e.g., in terms of body mass index) or quick policy changes, which is often described as a long-term process of* “muddling through”* [69]. Such findings might be observed, but in the long term, beyond the four-year timeframe of most politicians. To overcome these obstacles, it seems important to create short-term wins and implement advocacy strategies such as benchmarking [70], mobilizing citizens [71], media exposure [72]. Such efforts combined with awareness raising initiatives by the PHS, JOGG, or WHO may help in reframing childhood obesity as an involuntary, universal, and knowingly created risk and create engagement of the nonhealth sectors [39].

### 4.1. Strengths and Limitations

This study provided in-depth empirical data collected among local officials within health and nonhealth sectors in the Dutch context. Data representing the views from multiple policy sectors were urgently needed since few previous studies (e.g., [36]) captured these perspectives [45]. Although a modest number of officials were interviewed and we did not interview the same amount of policymakers in each case, their insights may provide policymakers and practitioners clues to overcome these obstacles when pursuing integrated PA policies. However, we acknowledge that we could have increased the amount of interviews and thereby make our findings more representative. Furthermore, our data mainly represents “large” local governments which had the intention to develop integrated PA policies. We acknowledge that large municipalities are a minority of the total amount of Dutch municipalities (40 out of 396). However, since ISC within large municipalities seems more pervasive than in smaller municipalities [34] and the problems they encounter (e.g., overweight, low SES) are more extensive compared to middle-size or small municipalities, this focus seems appropriate. The fact that we only included municipalities that intended to develop PA policies to prevent obesity might also have caused a bias towards more favorable attitudes towards PA policies and explain why none of the interviewees expressed doubts of the efficacy of PA to prevent or reduce obesity. Moreover, we applied a new theoretical perspective which might contribute to theory development since our findings might help to sensitize the OBs explored in this study. The “framework approach” based on the BCB provided a useful structure for data analysis and allowed us to obtain a broad view on obstacles. It is expected the BCB may also provide other researchers a way to compare results across studies and reflect upon their data. We acknowledge that applying such a deductive analysis has limitations with regard to capturing unexpected findings [58]. Therefore, we tried to remain open for unexpected findings by ongoing reflection; after each interview and during data analysis the two researchers (Anna-Marie Hendriks and Jolanda M. Habraken) reflected and compared results across three cases. To further stimulate contextualization of findings we are now comparing our findings with the outcomes of a document analysis.

## 5. Conclusion

Local governments offer a unique arena to develop integrated PA policies. However, to stimulate them to use this arena, more top-down support for local officials is needed during this “wicked” bottom-up innovation process. The process towards more integrated PA policies is expected to improve by focusing on boundary-spanning skills, adaptive management, and incentivizing process outcomes. Operational officials can enable tactical and strategic level officials to focus on this by providing feedback during the process. Furthermore, governmental leaders are recommended to broaden their outlook and seduce not only partners* outside* the government to collaborate but also partners* within* governments. Actors outside government, such as researchers and PHS, can assist in this process by reframing obesity and PA in the terminology of the nonhealth sector and show how an analysis of childhood obesity determinants translates into integrated PA policy.

## Figures and Tables

**Table 1:** Interview sample.

Interviewee's policy sector	Municipality	Interviewee's function
Interviewee 1, public health	Case 1	Policy advisor and process manager (operational level)
Interviewee 2, public health	Case 1	Policy advisor (operational level)
Interviewee 3, nature	Case 1	Policy advisor (operational level)
Interviewee 4, sports	Case 1	Policy advisor (operational level)
Interviewee 5, neighborhood work	Case 1	Policy implementer (operational level)
Interviewee 6, spatial planning	Case 1	Policy advisor (operational level)
Interviewee 7, education	Case 1	Policy advisor (operational level)
Interviewee 8, public health	Case 2	Policy advisor (operational level)
Interviewee 9, sustainable environments	Case 2	Policy advisor and program leader (tactical and operational level)
Interviewee 10, municipal strategy	Case 2	Policy advisor (operational level)
Interviewee 11, environment	Case 2	Policy advisor and program leader (tactical and operational level)
Interviewee 12, youth and education	Case 3	Policy advisor (operational level)
Interviewee 13, council member interested in public health	Case 3	Politician (strategic level)
Interviewee 14, public health service/centre youth and family	Case 3	Policy advisor (operational level)
Interviewee 15, spatial planning	Case 3	Aldermen (strategic level)
